# Strategies to support older adults’ mental health during the transition into residential aged care: a qualitative study of multiple stakeholder perspectives

**DOI:** 10.1186/s12877-022-02859-1

**Published:** 2022-02-24

**Authors:** Meg Polacsek, Marta Woolford

**Affiliations:** Benetas, Level 1, 789 Toorak Road, Melbourne, Vic 3123 Australia

**Keywords:** Aged care, Anxiety, Depression, Mental health, Transition

## Abstract

**Background:**

The move from home into residential care is one of the most stressful life experiences for older adults. ‘Relocation stress’ is a significant risk factor for anxiety and/or depression in aged care residents. Whether long-term or recently diagnosed, these mood disorders are associated with a decline in overall well-being, daily functioning and independence. The mental health needs of older adults are often poorly recognised and supported, including during the transition into residential care. Despite growing interest in how to facilitate this major life transition, few studies have taken the perspective of multiple stakeholders. The aim of this study was to explore resident, relative and staff experiences of the transition into residential aged care, and to identify strategies to support the mental health of older adults at this time. The role of pastoral care practitioners to facilitate transitions and support residents’ mental health was also examined.

**Methods:**

This phenomenological study involved individual interviews with 35 aged care residents, relatives and staff, between January and April 2021. Participants were selected using purposive sampling. Audio-recorded interviews were transcribed verbatim and supported by field notes. Data analysis followed Giorgi’s steps for qualitative data analysis.

**Results:**

Results were distilled into three main categories related to the overall transition experience, recognising and responding to residents’ mental health needs, and tailoring support to individual needs. A novel contribution of this study relates to the need to address a broad misunderstanding of the role of pastoral care and subsequent under-utilisation of a potentially valuable resource.

**Conclusions:**

By describing transition experiences and the resources to support residents’ mental health, findings of this study provide practical, ‘real world’ suggestions for reducing relocation stress. New resources developed from the findings include guides, checklists and short question-and-answer films, in which current residents and staff describe strategies to support mental health and overall quality of life. Similar resources could be used to support transitions in other care settings.

## Introduction

Defined as passages ‘from one life phase, condition or status to another’ [1], transitions are experienced throughout life. They are often conceptualised as life ‘stages’, but each is an experience unique to the individual. Many transitions in older age are positive and welcomed, such as planned retirement, participation in volunteering and inter-generational activities, and travel [2, 3]. However, older adults also experience a range of negative transitions, of which the transition from home to residential aged care is a particularly significant one [4, 5]. Depending on the context, residential aged care may be known as ‘aged care’, ‘long-term care’ or ‘nursing home’. These facilities provide accommodation and personal care 24-h a day, with access to nursing and other healthcare, and social and emotional support.

‘Relocation stress’ is increasingly used to describe the effect of a transition into residential aged care on older adults, specifically regarding their mental health. Often used inter-changeably with ‘relocation stress’, the term ‘relocation stress syndrome’ has come to represent particularly strong responses to the transition into aged care, whereby the older adult experiences heightened confusion, anxiety, depression and loneliness [6, 7]. Relocation stress is associated with a significant decline in older adults’ mental health, making it a clear risk factor for anxiety and depression for older adults moving into aged care [8–10].

Globally, anxiety and depression are the most prevalent mental illnesses in older adults [11]. Whether long-term or only recently diagnosed, depression and/or anxiety in older adults are associated with a decline in overall well-being, daily functioning and independence, increased disability, suicidal ideation and mortality [12–14]. There are also indications of a reciprocal link between depression and cognitive impairment, including dementia [15, 16]. Across countries and cultures, major life transitions serve as some of the most significant determinants of depression and/or anxiety in older adults [4, 17].

The prevalence of depression and/or anxiety in older adults living in the community tends to decline with age [18, 19]. However this is not the case in residential aged care [20]. In a study of the prevalence and determinants of mental illness in residential aged care, Amare et al. [14] found almost 58 per cent of residents had at least one diagnosed mental illness, of which depression (46 per cent) was the most common. According to the Australian Institute of Health and Welfare [19], up to 75 per cent of aged care residents have symptoms of depression. Similar differences in estimates exist for anxiety, with studies reporting 15 per cent of residents in their study had a diagnosis of anxiety [14], while others report ranges between three and 20 per cent [20].

Ageing and clear evidence of the deleterious effect of admission to residential aged care on mental health confirm the need for practical advice and interventions to support older adults’ through this major transition [21]. Despite the availability of validated psychological interventions for aged care residents, access to specialist mental health support, including psychologists, in this setting is often poor [22, 23]. More common applied examples of evidence-based approaches to supporting individual residents’ mental health include facilitating new friendships, providing meaningful activities [8], bereavement support, family and peer support [21], and pastoral care or chaplaincy services [23, 24]. The potential of pastoral care services was of particular interest in the current study, as the participant organisation—a leading faith-based provider of aged and community care in Australia—employs a team of specialist non-denominational pastoral care practitioners to provide individualised and ongoing support to older adults and their relatives.

With qualifications and experience in theology, social sciences, grief support and/or palliative care, it is the role of pastoral care practitioners to provide emotional, spiritual and social support [25, 26]. Based on trusting relationships, these practitioners may serve the role of confidante, companion or spiritual guide [25]. Despite favourable evidence for supporting mental health, however, the take-up of pastoral care is often limited by poor understanding by residents and staff of the role of pastoral care practitioners [24]. Common misconceptions relate to pastoral care serving a religious function [26], or not making a connection between spiritual and emotional support, and mental health [27]. Yet there is a strong, reciprocal relationship between the same and different elements of spirituality, emotional wellbeing, mental health and overall quality of life [27, 28]. Thus, we submit that on-site pastoral care practitioners are well placed to support older adults in their transition into residential aged care.

The aim of this study was to explore resident, relative and staff experiences of the transition into residential aged care, and to identify strategies to support the mental health of older adults at this time. The potential or actual utilisation of pastoral care practitioners to facilitate resident transitions and support their mental health was also examined.

## Methods

### Design

A qualitative approach to data collection and analysis was used, following the principles of phenomenological research. By placing the emphasis on participants’ subjective (‘lived’) experiences of transitions, aged care and mental health [29], multiple stakeholder perspectives were collected.

Approval by a human research ethics committee was obtained prior to data collection (Monash University Human Research Ethics Committee Project ID: 26,929). Written consent was obtained from each participant, and participants were informed of their right to withdraw from the study at any time (none withdrew).

### Setting and sample

The study was conducted in the residential division of a major not-for-profit aged care provider in the Australian state of Victoria. The organisation provides services along the continuum of care, from primary health care to home-based support, retirement living and residential aged care. To recruit a diverse sample, four residential homes—two in metropolitan and two in regional areas—were selected as sites for the study. Purposive sampling was used to recruit residents and staff who (i) were currently living or working in one of the four homes and (ii) could provide informed consent. Staff participants were also recruited from the organisation’s head office. Exclusion criteria for residents were if they were unable to provide informed consent, either from cognitive impairment or from other significant health issues; had moderate to severe cognitive impairment that prevented meaningful participation; were currently in respite care; or had transferred from an acute setting (that is, hospital and not from home). Excluding those transferring from an acute setting aligned with the host organisation’s function of supporting older adults living in the community, who may at a later stage enter residential aged care. This decision was also supported by a review of the literature, which revealed a gap in the evidence to support non-hospital transitions into residential care [30, 31]. Relatives of current residents and volunteers were also invited to participate.

### Recruitment

Recruitment of residents comprised of (1) researchers visiting each of the four residential homes and inviting residents to participate; and (2) placing flyers on common area notice boards (such as in dining rooms and recreation areas). These flyers provided information on the study and invited those who were interested to contact the researchers directly. The researchers also asked residential staff to identify residents who might be interested in participating. To avoid the pitfalls of gatekeeping, whereby staff may try to guide the recruitment of particular individuals [29], the researchers asked only if there were any residents who should not be approached, if they did not meet the inclusion criteria. Prospective resident participants were approached in the privacy of their own rooms.

For staff participants, recruitment involved placing an advertisement in a regular internal staff e-newsletter, word-of-mouth by residential managers to staff and volunteers, placing flyers on communal noticeboards, and sharing information on the study in residential staff team meetings. Those who were interested in participating contacted the researchers, who explained the research in plain English, referring to the appropriate explanatory statement, and giving adequate time for questions. This information stated the voluntary nature of participation and assurance that the decision to participate, decline to participate, or withdraw from the study could be made at any time, with no explanation needed, and no negative consequences. Participants were free to refuse to answer any questions, with no explanation needed. Any arrangements to participate were made directly with the researchers, and managers were not involved in any decision-making for members of their staff.

### Data collection and analysis

Data collection and analysis took place between January and April 2021. Data were collected through individual interviews, with parallel interview guides constructed for residents, relatives, staff and volunteers. All interviews commenced with the researchers obtaining basic demographic information, limited to age, sex and cultural background. Interview questions were informed by findings from a previous ‘quality of life’ project conducted by the organisation, and with reference to relevant literature [32, 33]. Questions focused on the experience of the transition into residential aged care from participants’ different perspectives, including whether the transition was planned or unplanned; the level of resident involvement in decision-making, and the timing of the transition. More specific questions related to residents’ mental health, with all participants asked to suggest practical strategies to support resident wellbeing (Table 1).

**Table 1:** Sample of interview questions

Questions for all participants	Questions for residents and relatives	Questions for staff
• Could you describe what information you might want, to help prepare for residential care?	**• **Can you describe what it was like planning the move?	• If you were to put yourself in a resident’s shoes, what sort of things do you think would influence the experience, positively or negatively?
**• **What was it like for you/them, when they moved in? What sort of help did you/they get to settle in?	**• **Could you describe the most useful information you received before the move?	• How could you, in your role, help to reduce residents’ ‘relocation stress’?
**• **Can you tell me about the most challenging part of a move into residential care?	**• **Were you/they given any information or support about looking after their mental health?	• How might you respond if a resident raised concerns about their mental health?
**• **If you were to give someone else advice about moving into residential care, what are the main points you’d make?	**• **What information or strategies do you think might help to reduce any anxiety, depression, loss or loneliness associated with the move?	• Could you describe any additional support that may reduce residents’ anxiety or depression, or any sense of loss or loneliness?

All interviews with residents, relatives and volunteers were conducted face-to-face. Most staff interviews were conducted in person, with some held by telephone and/or video. Each interview took approximately 45 min. To confirm participant understanding and avoid misinterpretation, researchers undertook ‘member checking’ by summarising, repeating and paraphrasing participants' words or actions at several points during and at the end of the interview [34]. By increasing the accuracy of participants’ views, this practice supports trustworthiness in the data [29].

Recognising the role of context in qualitative research, observations undertaken at residential homes were recorded in detailed field notes. These notes were included as a component of the overall data, adding depth to the researchers’ understanding of the influence of the physical environment on participants’ experiences [35]. Field notes also supported the researchers in their reflections and helped to reduce bias in data collection and analysis [35].

Data analysis followed Giorgi’s [36] steps for qualitative data analysis, whereby both researchers read and re-read the transcripts and interview notes, to understand the ‘whole’. Initially, the researchers worked independently to identify ‘meaning units’, before coming together to transform the meaning units into common themes.

This flexible, data-driven approach enabled an understanding of participant experiences—Giorgi’s [36] ‘structure of experience’—and allowed themes to be applied across participant groups and settings. Participants’ own words or phrases were used to illustrate and give meaning to the data. This practice supports the credibility of findings, as participants’ meanings are accurately represented in a way that ‘gives life’ to the study [29]. For data analysis and reporting, pseudonyms were allocated to all participants.

## Results

A total of 35 interviews were conducted, including 14 residents, 19 staff, one relative and one volunteer. Residents’ average age was 84.9 years (range: 70 to 92 years), most were female (*n* = 8, 57%) with an average residential aged care stay of 18 months.

Staff participants comprised residential managers (*n* = 3), personal care workers and nurses (*n* = 5), ‘lifestyle’ staff (*n* = 2), staff from quality and customer service teams (*n* = 5), and pastoral care practitioners (*n* = 4). All staff participants had direct experience with managing or supporting older adults’ transition into residential care. Their average age was 36.6 years (range: 30 to 62 years) and most were female (*n* = 17, 89%).

The shared focus of interviews facilitated the collation of all participants’ responses into three broad themes, representing the overall experience of the transition, the importance of recognising and responding to residents’ mental health needs and tailoring support to individual needs. Participants’ experiences and suggestions informed the co-development of practical strategies and resources.

### The overall transition experience

In the first theme, resident participants considered their overall experience of moving into residential aged care. While participants across all groups acknowledged the significance of the move, residents considered their role in the decision-making process, and accepting the need to move to aged care, as critical to a successful transition.

I wanted to come … I like it, I am happy (Jacquie, resident, 70 years).

I decided to go to aged care. This home is the one I chose first and then all of a sudden I was offered a spot. It was smooth, there were no problems (Oliver, resident, 91 years).

Conversely, resident participants who had little time to consider their options, were not involved in the decision-making process or were reluctant to move were less likely to report their contentment with the experience.

It’s a slow process, especially for people who didn’t want to come (Josie, resident, 88 years).

I realised I was going down and would be needing help soon … [but] I can’t remember being told anything [about the move] … that made it harder for me … it’s not the best thing, to be suddenly brought here (Ingrid, resident, 88 years).

Whether the transition had been voluntary or involuntary in nature also had a clear influence on how the residents had come to terms with the move.

We know the effect of an unplanned transition, or a rushed transition … it’s detrimental to people's mental health (Mike, nurse).

While a significant amount of paperwork was required for the resident’s transition, most participants identified a need for ‘real world’ information to help residents settle in. Real world information, such as descriptions of daily routines and expectations, was identified by the participating relative as instrumental to helping them support their family member through their transition. Participants explained this information should be given before moving into the home.

It would be handy to be given some sort of booklet … some sort of introduction to the place. Nobody sits down with us to tell us what we can do or get (Sarah, resident, 88 years).

[I’d want to know] what the ‘rules’ are in terms of staff supporting me? Should I expect staff to knock on the door before they come in? Who do I go to if I've got a concern or if I'm not comfortable with something? (Gail, quality manager).

These suggestions resonated with residents, who described difficulties getting used to the new environment and routine. For some, it was evident that the uncertainty associated with the move was stressful and confusing, particularly in the initial post-transition period.

It’s all a challenge when you are new. It takes a bit getting used to … everything is difficult (Yuri, resident, 87 years).

Living to a schedule instead of doing what I like … I am used to it now, but it would have been good to know (Sarah, resident, 88 years).

Someone from the home should tell new residents about the routines and activities (Cathy, resident’s daughter).

Asked what might help new residents settle in, participants confirmed the importance of meeting people and making new friends. New friendships were also a way to get used to the new environment and routine.

I think it is important to promote friendships and relationships, because it gives people the opportunity to have that peer respect, but also the ability to have someone to talk to, or to ask questions (Meryl, pastoral care practitioner).

While making new friends in the home usually reduces residents’ sense of loss and loneliness, most participants acknowledged a gap in how residents were introduced to each other.

We no longer get introduced to new people, which is a shame. It’s nice to be introduced to new people (Alice, resident, 90 years).

We need to improve how we introduce residents (Sheila, pastoral care practitioner).

Staff participants also explained that delayed or limited information about the arrival of new residents, and information about them, reduced their efforts to support the transition.

Sometimes I’m not told that a new resident has arrived … it’d be nice to have a bit more of a procedure or a plan in place, so we don't let people come in and feel like they're not supported (Emma, pastoral care practitioner).

I’m not sure what information they [residents] get, or how prepared they are. And we don’t know about the resident and their needs until they arrive … it would be good to get something [information] from them [before they arrive] (Penny, personal carer).

Where circumstances allowed, there were clear benefits associated with personalising a new resident’s room before they arrived. Participants described how comforting it could be for residents to have something familiar waiting for them.

If I had a bit of notice, I would ask the family to start bringing stuff in before they moved in. So, I would put photo frames up, some cushions from home, I'd have their favourite music playing in the background (Pauline, quality manager).

It doesn’t always happen, but I always like to see it when families can come in and set up the room before the person comes. I know, myself, that my own pillow and blanket are so comforting … having something there that is very comforting to you (Sheila, pastoral care practitioner).

My daughters set up my room. They brought my things before hand and helped me settle in … it was good (Jacquie, resident, 70 years).

### Recognising and responding to mental health needs

In the second theme, participants gave their different perspectives on how they might recognise or respond to signs of depression and/or anxiety in residents. For staff, knowing about a new resident’s pre-existing mental health condition before their arrival allowed them to set up appropriate strategies to support the person, often in collaboration with family members. It was also important to be given timely and accurate healthcare information, including background on the person’s past experiences and triggers.

If we know from the get-go [start] that the person is feeling quite depressed or anxious, maybe the family can help us understand what might be helpful (Sheila, pastoral care practitioner).

We need to find out who the resident has seen for their [depression]. Can we get any copies of reports to try and understand? We don't know what the triggers are. How can we care for someone if we don't really understand what they’ve been through? (Jenny, residential manager).

Clinical care staff and residential managers identified the importance of being proactive in recognising and treating mental health issues. Despite this, staff participants working directly with residents reported a need to improve this process, with evident gaps in practice.

Mental health is often overlooked … and the pathway to receiving mental health assessment and services needs to improve (Tina, residential manager).

Mental health [treatment] has lacked in aged care for a long time. We had a man, he was depressed, and there was no support for him (Penny, personal carer).

Managers and pastoral care practitioners, in particular, reported that they would refer to doctors or mental health professionals for support.

If [a resident] was suffering with depression or anxiety, I think it would be very helpful to them to have somebody [mental health professional] there (Sheila, pastoral care practitioner).

If somebody has a mental health diagnosis, then we should be thinking about what we need to put in place for them (Tina, residential manager).

For residents, mental health was often related to feelings of loss and loneliness. Often, they first sought support from relatives and other residents.

I had a sense of loss, but I talked to my family … the help from family is very good (Jim, resident, 92 years).

During lunch or dinner, we do notice each other … we notice things, if people are having a problem. Then we talk about it (Sarah, resident, 88 years).

[You need] a friend who can help if you’re getting depressed (Josie, resident, 88 years).

If they felt it was appropriate, residents would encourage others to seek help from the staff. Some had received professional support for their mental health.

I’d tell a team member if I think a resident is depressed (Josie, resident, 88 years).

A few times, I’ve had a bit of a ‘melt-down’ … the staff have been really good. The nurses come in and I can talk with them (Sarah, resident, 88 years).

Despite the evidence in favour of pastoral care support in aged care [24], it was clear that this resource was under-utilised, with just one resident accessing support from the pastoral care team.

I’d tell them to talk to somebody … pastoral care is a good start. I talk to them quite often (Jim, resident, 92 years).

### Tailoring support to individual needs

For residents, a sense of being recognised and treated as an individual cushioned the experience of the transition. Most often, this was illustrated by statements that demonstrated that they could set their own routines and pursue their own interests. While not articulated as such, these strategies are known to support mental health.

I’ve kept my individuality … I can continue my routines, leave [for excursions], help out … [that was] a pleasant surprise (Alistair, resident, 87 years).

I love gardening. I saw no-one was taking effort [looking after] the garden, so I asked if I could fix it. I brought a lot of my plants here (Oliver, resident, 91 years).

Similarly, staff participants highlighted the importance of responding to each resident’s individual needs and preferences.

[Residents] want the focus to be, ‘This person truly knows about me, they truly care about me, and they're interested in me’ (Mike, nurse).

Their individuality is important for any transition. I'm a great believer in knowing the history of someone … understanding where there may be speed bumps [challenges] along the way (Jonathan, clinical care coordinator).

Staff described the benefits of knowing as much as possible about the person before they move into the home. This would allow them to focus less on the formal process of the admission and more on the new resident’s individual needs.

The nurses put photos up, brought in air-conditioning … I really like that, makes it feel more like home. It *is* home now (Yuri, resident, 87 years).

While processes were in place to manage the transition, potential improvements to practice were identified.

It's not just a phone call, a booking, people filling out paperwork and coming and having a quick look at the room … there's a big piece that follows on from that … making people feel safe and comfortable, how things work here (Gail, quality manager).

Reflecting on existing resources to support residents’ mental health, participants recognised a need to address a common misconception regarding pastoral care. Most resident participants reported confusion or scepticism about the underpinning philosophy and function of pastoral care, while those staff who understood the potential value of pastoral care to support residents often used another term to describe the service.

I don’t want to be bible bashed [persuaded to believe] by anyone! (Pete, resident, 77 years).

I avoid mentioning pastoral care … it’s too religious (Barbara, lifestyle coordinator).

For the generation [residents] here, ‘pastoral care’ means church, it means religion … for some people, it’s a big wolf [frightening] (Julia, pastoral care practitioner).

The tendency for pastoral care to be misunderstood frequently resulted in the under-utilisation by residents of a freely available resource, and was frustrating for pastoral care practitioners who were keen to engage with and support residents throughout their transition.

I’ve been here for five years, so people know me as a person, but my title confuses people. It’s a continual teaching and reminding them [what I do], especially when new staff come on board (Sheila, pastoral care practitioner).

I actually sent out an email saying, ‘Pastoral care is not what you think it is’. Basically, just say, ‘Pastoral care is about your wellbeing … it's a holistic view … it is not based on a religious view’ (Julia, pastoral care practitioner).

For care staff, there was value in enlisting additional pastoral care support at the time of a new resident’s arrival.

There's a lot to do [when a new resident arrives], but it's all the clinical stuff. I don't understand why pastoral care aren't involved right from the start … [new residents] need a friend (Diane, clinical care coordinator).

Pastoral care would help with the transition of our residents … if we could have someone follow them each day of the transition that would be amazing (Holly, nurse).

The potential of a ‘buddy system’ was raised by several participants, with current residents considered a largely underutilised resource in providing information and support to new residents.

The best thing would be to have a buddy … a welcoming buddy … [and] whatever they [new residents] ask, you would know the answer (Josie, resident, 88 years).

[A buddy system] could work here, because the residents here like to be productive and helpful … I think that they would really love to be a part of something like that (Sheila, pastoral care practitioner).

Potential obstacles to a buddy system were considered, largely by staff, including the different needs and personal capacity of residents. The potential of volunteers to perform this role was raised, as was the need for clear guidelines and processes.

We've done a buddy system, but it can be hard, depending on your demographic in the home … most of the homes are just too high level [of care needs] (Pauline, quality manager).

With the buddy, you need to make sure they are not ‘gate keepers’ … make sure it’s not overdone (Roger, resident, 76 years).

Volunteers could take on the role of a buddy, but this would require significant investment into policy and process updates, and central oversight … you want to make sure that the resident gets the right information about what goes on at the facility (Jenny, residential manager).

## Discussion

Although transitions occur over time, they are often triggered by an event or experience that precipitates the change [13, 37]. Admission to residential aged care often follows a crisis, such as a fall at home or hospitalisation [8]. With little time to prepare, older adults are often marginalised in the decision-making process and may not know that the admission is permanent [38]. Hence, the transition into residential aged care is considered one of the most stressful life experiences for older adults [8, 39].

The overall aim of the current study was to understand and support the mental health needs of older adults transitioning from home into residential aged care. All participants consistently acknowledged new residents’ vulnerability to depression and/or anxiety, in particular, and highlighted the need to improve recognition of and response to symptoms. Conceptualised under three inter-related themes, participants considered the overall transition experience, the importance of recognising and responding to residents’ mental health needs and tailoring support to individual needs.

Whatever the circumstances, the move into residential aged care is an emotional and often stressful event [21, 40]. At best, the older person makes an informed decision to enter residential aged care, maximises the opportunities available to them to form friendships and engage in meaningful activities, and receives high quality care. More common, however, is a reluctant transition that is beset with challenges [4, 5, 21]. In the current study, all participants acknowledged the influence of the decision-making process on the transition experience. A significant determinant was whether the move was voluntary or involuntary. Across different population groups and contexts, evidence has shown that voluntary transition is associated with positive outcomes, including gratitude, a sense of autonomy, acceptance (no regrets) and improved quality of life [13, 41]. Conversely, regret, loss and anger are more commonly experienced by those for whom a transition into residential aged care was involuntary, with a similarly traumatic effect on their relatives [41]. Adverse consequences for residents entering aged care under these conditions include depression, anxiety, increased confusion, disorientation and isolation, and higher rates of morbidity and mortality [4, 6, 8, 21]. Global population ageing and a commensurate increase in older adults living with depression and anxiety require concerted efforts at recognising and respecting the experience of transitioning into residential aged care [4]

Regarding strategies to help residents plan their transition and settle into the residential home, more ‘real world’ information is needed to inform new residents of typical routines, visitor arrangements, staff roles and responsibilities, and facilities in and outside the home. Notwithstanding the fundamental importance of documenting resident’s clinical history and care needs, there is a clear need to improve how daily life is described to new residents, and to enlist individual support to help them adjust to their new home. The findings from the current study have informed improvements to the overall resident admission process, from a more structured approach to residential tours (COVID-permitting), to a comprehensive review of the resident handbook. For tours by prospective residents and their relatives, a new checklist will provide prompts for questions on how individuals’ social, physical, spiritual and cultural needs can be met. A staff version of the tour checklist guides the conduct of the tour. The next iteration of the resident handbook will provide more information to support the transition and detail the practical aspects of life in the home. For example, residents in the current study wanted to know their right to privacy in their own room, whether meal times were flexible, and if they could leave the premises. Another new resource is a diagram illustrating a ‘typical day’ in residential aged care, with indicative times for meals, activities, laundry and staff shift changes.

In theory, the onus is on service providers to support a positive transition [12] and ‘cultivate a sense of home’ [41]. In practice, however, this responsibility is often not delegated to an individual member of staff. Rather, the new resident encounters a wide array of new people, whose roles may not be made clear to them, and whose availability or presence may be inconsistent [42]. This makes it difficult for residents to get to know the care staff [43], and may limit their opportunities for early introductions to other residents. Given their availability in the current context, there is clear opportunity for pastoral care practitioners to bridge this gap, as they are well placed to welcome and develop relationships with residents, and provide emotional support.

An additional benefit of developing supportive relationships with pastoral care practitioners is that they are not part of the direct care team [25]. They are generally less constrained by the demands on care staff to provide personal and clinical care, and can take their time to create a safe space in which emotional, spiritual and social support may be sought and received [25]. In the current study, the main barrier to using pastoral care related to the common misconception that it was limited to religious or spiritual support. Scepticism about the role and purpose of pastoral care practitioners results in the under-utilisation of a potentially valuable resource [24, 25]. A direct outcome of the current study is a review of the name, role and promotion of pastoral care services in the organisation, with a view to improving access by residents and referral by staff to this valuable resource. Opportunities for facilitating a ‘buddy system’ could also be maximised, with potential benefits to new and current residents, and staff [12, 44].

To ensure that the content and format of these new or revised resources meet their needs, a consumer-led approach involving older adults, their relatives and staff was followed [45]. Through this process, residents shared their explicit preference for short question-and-answer films. Fortunately, the study budget allowed for the production of two short films, in which current residents, relatives and employees shared their experiences and suggestions regarding residents’ mental health. These films are being embedded into the pre-admission process and are available on the organisation’s website.

The study has certain limitations. As a qualitative study in which purposive sampling was used to recruit participants from one large multi-centre aged care provider in Australia, the findings may not represent the views of residents, relatives and employees in different settings. In addition, COVID-19 restrictions in place at the time affected recruitment of relatives and volunteers, with tighter controls over the number and frequency of visitors to the homes. As a result, only one relative and one volunteer participated in the study. Although saturation of participant data was reached, the study may also have benefited from the participation of other stakeholders. Given the timing of the project, we have not yet been able to determine the extent to which the new resources have been adopted in practice. Finally, we did not interview residents with moderate to severe dementia. In future, it would be useful to enable the meaningful involvement of a broader range of stakeholders, including people with dementia, in studies such as this.

It is also worth noting that, although it was clearly a stressful time, no participants in the current study used the term ‘relocation stress’ to describe the effect of the transition on residents’ mental health. There is a risk that this apparent gap between academic (or policy) terminology and ‘user experience’ may impede timely and accurate recognition and response to symptoms of relocation stress by older adults, their relatives and the staff who look after them. People who are unfamiliar with a term may be missing out on information or support to help them manage the issue [46]. Consistent, clear language may facilitate understanding and the subsequent recognition of and response to residents’ mental health, particularly when it aligns with the words used by older adults themselves [38]. This is particularly important in this cohort, because relocation stress manifests differently among residents [4, 39].

## Conclusion

Across countries and cultures, the transition from home into residential aged care is one of the most significant determinants of depression and/or anxiety in older adults. The results of this study support previous findings, but add new insights for practice. By describing transition experiences and suggestions for support, the findings of this study have practical implications for reducing relocation stress in older adults entering residential aged care. The resources developed with participants in the current study could be adapted to inform improvements to practice in other care settings, such as between hospital and residential aged care, or from independence at home to receiving home care services. A novel contribution of this study relates to the need to address a broad misunderstanding of the role of pastoral care and subsequent under-utilisation of a potentially valuable resource.

## Data Availability

The dataset for this study is not publicly available, as there is a risk of compromising the individual privacy of participants. However, de-identified parts of the interview transcripts may be obtained from the corresponding author, upon reasonable request.
